# Versatile MXenes for Aqueous Zinc Batteries

**DOI:** 10.1002/advs.202305806

**Published:** 2023-11-20

**Authors:** Huan Liu, Zijun Xin, Bin Cao, Bao Zhang, Hong Jin Fan, Shaojun Guo

**Affiliations:** ^1^ College of Materials Science and Engineering Xi'an University of Science and Technology Xi'an 710054 China; ^2^ School of Physical and Mathematical Sciences Nanyang Technological University Singapore 637371 Singapore; ^3^ School of Materials Science and Engineering Peking University Beijing 100871 China

**Keywords:** aqueous zinc‐ion batteries, cathodes, electrolytes, MXenes, zinc anodes

## Abstract

Aqueous zinc‐ion batteries (AZIBs) are gaining popularity for their cost‐effectiveness, safety, and utilization of abundant resources. MXenes, which possess outstanding conductivity, controllable surface chemistry, and structural adaptability, are widely recognized as a highly versatile platform for AZIBs. MXenes offer a unique set of functions for AZIBs, yet their significance has not been systematically recognized and summarized. This review article provides an up‐to‐date overview of MXenes‐based electrode materials for AZIBs, with a focus on the unique functions of MXenes in these materials. The discussion starts with MXenes and their derivatives on the cathode side, where they serve as a 2D conductive substrate, 3D framework, flexible support, and coating layer. MXenes can act as both the active material and a precursor to the active material in the cathode. On the anode side, the functions of MXenes include active material host, zinc metal surface protection, electrolyte additive, and separator modification. The review also highlights technical challenges and key hurdles that MXenes currently face in AZIBs.

## Graphical abstract

MXenes are being broadly explored for applications in aqueous Zn metal batteries. This review highlights the versatile functions include active cathode material or 2D conductive additive, Zn host materials or surface protection, 3D framework, flexible support, and separator modification. It is concluded that the applications of MXene in Zn metal batteries have not been fully explored. Technical challenges and opportunities are underlined at the end.

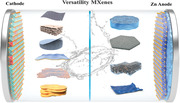

## 1 Introduction

With the extensive utilization of fossil fuels and ever‐growing energy demands in recent years, renewable energy systems (electrochemical energy storage, solar energy, wind energy, and tidal energy) are being recognized as promising technologies to mitigate environmental pollution and energy scarcity. Electrochemical energy storage is regarded as an attractive energy storage solution due to its stability and environmental eco‐friendliness.^[^ ^1^ ^]^ Portable and mobile devices are predominantly powered by lithium‐ion batteries (LIBs), due to their superior energy density and extended service life.^[^ ^2^ ^]^ However, the finite reserves, uneven distribution and unstable cost of lithium, and the safety risk posed by volatile and flammable organic solvent‐based electrolytes are common difficulties in advancing LIBs.^[^ ^3^ ^]^ Additionally, other emerging battery technologies such as sodium‐ion and potassium‐ion batteries offer advantages in terms of cost due to the abundant sodium and potassium resources and matured manufacturing process. Although substantial progress has been made in the development of electrode materials and electrolytes for both types of emergent batteries, their safety issue associated with organic electrolytes remains a concern, similar to LIBs.^[^ ^4^ ^]^

Rechargeable batteries that use aqueous electrolytes are regarded safer than their nonaqueous counterparts. Here multivalent charge carriers including Mg^2+^, Al^3+^, and Zn^2+^ and abundant alkali metal cations such as Na^+^ and K^+^ are being used for aqueous batteries. In particular, aqueous zinc‐ion batteries (AZIBs) are under spotlight of current research. An AZIB device consists of a zinc metal anode, a mild neutral pH (or slightly acidic) electrolyte, and a host cathode designed to accommodate Zn^2+^ ions are in the spotlight. In addition to the high theoretical capacity (820 mAh g^−1^) of zinc metal, the low‐cost and high‐safety aqueous electrolyte system make AZIB more favorable for large scale energy storage when compared to currently used alkali metal‐ion batteries with organic electrolytes.^[^ ^5^ ^]^ Cathode materials and zinc metal anodes, including their interfaces with electrolytes, have been the focus of AZIB research.^[^ ^6^ ^]^ Several potential cathode materials, including manganese (Mn)‐based oxides,^[^ ^7^ ^]^ vanadium (V)‐based oxides,^[^ ^8^ ^]^ Prussian blue analogues (PBAs),^[^ ^9^ ^]^ polyanionic compounds (phosphates, fluorophosphate, etc.)^[^ ^10^ ^]^ and Co‐based oxides^[^ ^11^ ^]^ have been explored. The Mn‐based materials are widely investigated due to their high theoretical capacity of 308 mAh g^−1^, achieved through single‐electron transfer between Mn^4+^ and Mn^3+^, high voltage (≈0.8–1.8 V).^[^ ^12^ ^]^ The V‐based materials with multivalence state V (from +5 to +2) and large‐tunnel framework benefit accommodation of Zn^2+^ ions.^[^ ^13^ ^]^ Although their average discharge voltage is low, ≈0.8 V, their discharge capacity frequently exceeds 300 mAh g^−1^.^[^ ^14^ ^]^ However, owing to their poor intrinsic electrical conductivity and dissolution of metal ions during the phase transformation with the intercalation of zinc ions, both Mn and V‐based oxides demonstrate inferior rate capability and limited cycle life.^[^ ^15^ ^]^ In addition, notwithstanding their noteworthy gravimetric capacity of 820 mAh g^−1^ and volumetric capacity of 5855 mAh cm^−3^, zinc anodes tend to form dendrites and undergo hydrogen evolution during charge and discharge cycles. This can lead to serious issues such as battery short, internal swelling, and the low utilization of the zinc anodes.^[^ ^16^ ^]^ Zinc dendrites are formed due to the non‐uniform distribution of the electric field and flow of Zn ions, while hydrogen evolution occurs as a result of the gradual corrosion of the zinc anode in aqueous solutions and its electrolysis during the stripping/plating process.^[^ ^17^ ^]^

In addition to electrodes, the aqueous electrolyte is of vital importance in AZIBs. This electrolyte has a significant impact on the reversible nature of zinc anode, along with the ion insertion chemistry of the cathode during charge/discharge process.^[^ ^18^ ^]^ Several strategies, such as guest pre‐intercalation (e. g. Na, Al, K, and La)^[^ ^19^ ^]^ and carbon (graphene,^[^ ^20^ ^]^ CNTs^[^ ^21^ ^]^ and porous carbon^[^ ^22^ ^]^) composite strategies for the cathode, and the host and zinc metal interface regulation technology, and highly concentrated “water in salts” electrolytes (21 M LiTFSI+1 M Zn (CF_3_SO_3_)_2_ electrolyte) for anode^[^ ^23^ ^]^ etc. have been explored to overcome the above issues.

**Figure 1:**
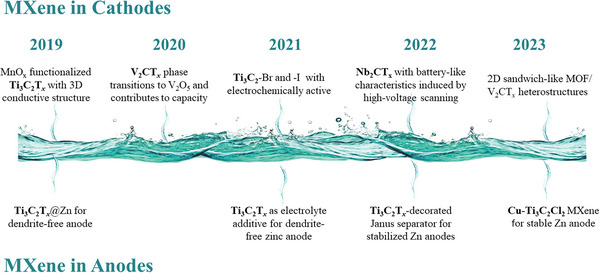
Timeline of MXenes and its derivatives for application in AZIBs.

MXenes have evolved as a versatile class of 2D materials that can be employed in many components of AZIBs, including as cathodes, anodes, electrolytes, and separators (**Figure** **1**). MXenes, which are transition metal carbides/carbonitrides/nitrides with a 2D structure, metallic conductivity, highly active surface, and tunable structure, have been investigated thoroughly in AZIBs.^[^ ^24^ ^]^ MXenes are produced mainly via selectively etching of “A” element from its layered ternary MAX precursor with a representative formula of M*_n_*_+1_AX*_n_*, where M denotes a transition metal (such as Ti, V, Zr, and Nb), A represents IIIA or IVA element (such as Al, Ga, Si, *etc*), and X signifies carbon and/or nitrogen. A general formula represents MXenes as M*_n_*_+1_X*_n_* T*_x_*, where T*_x_* refers to abundant surface functional groups (‐O, ‐OH, ‐F, ‐Cl, ‐Br, ‐I) where the nature of functionality varies with the etchant and etching procedure.^[^ ^25^ ^]^ Till now, over 40 different types of MXenes have been successfully synthesized, including Ti_3_C_2_T*_x_*, Ti_2_CT*_x_*, V_2_CT*_x_*, Nb_2_CT*_x_*, Mo_2_TiC_2_T*_x_* and Nb_4_C_3_T*_x_*.^[^ ^3^, ^24^, ^26^ ^]^

**Figure 2:**
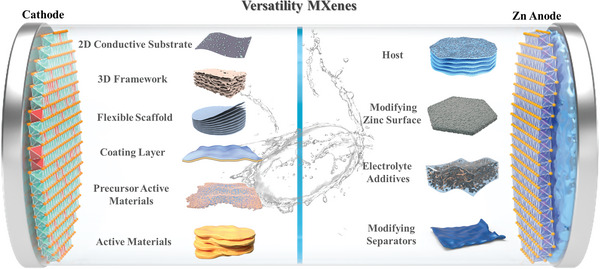
A summary of MXenes functional versatility in AZIBs.

The progress of MXenes in AZIBs has been remarkable, but a comprehensive review of their functional design aspects has been lacking in the past few years. In this review, we will examine the evolution and achievements of MXenes in cathode and anode for AZIBs, focusing on the functionalities and optimization strategies of MXenes‐based materials. **Figure** **2** shows a schematic representation of the role of MXenes in AZIBs and their progression. First, we discuss the applications of MXenes as a cathode in conjunction with zinc storage active materials, such as the 2D conductive substrate, 3D frameworks, flexible scaffold, and coating layer. Then, we comprehensively sum up a wide range of practical procedures, including surface functional group control, oxidation, and ion intercalation, through which MXenes and their derivatives may be employed as active materials and active materials precursors for zinc storage. Additionally, the pivotal role of MXenes in AZIBs anode is explored, encompassing their functions as hosts, modifiers of zinc metal surfaces, electrolyte additives, and separators, effectively mitigating dendrite formation and corrosion of the zinc anode. Moreover, future advancements in MXene utilization within AZIBs hold promising prospects for further development.

## 2 MXenes in AZIBs Cathodes

MXenes are considered competitive materials for AZIBs cathode due to the following reasons: 1) MXenes can operate as stable and conductive substrates and enhance the cycle and rate performance of active cathode materials (such as Mn, V‐based oxides) as a result of their layered 2D structure and high electrical conductivity; 2) The substrate for increasing zinc ion transport kinetics in composite cathode material may be modified 3D MXenes; 3) The superior mechanical flexibility permits the production of flexible wearable AZIBs; 4) In terms of specific capacity, voltage window, and cycle life, a wide variety of MXene derivatives and plentiful surface functional groups may differentiate AZIBs cathode. Three varieties of MXenes, namely Ti_3_C_2_T_x_, V_2_CT*_x_*, and Nb_2_CT*_x_*, have been explored as cathodes for AZIBs, including capacity, cycle stability, rate capability, and electrolyte utilized in AZIB systems. This section examines the prospects of MXenes as cathodes for AZIBs.

### 2.1 MXenes as a Conductive Substrate Support for Active Materials

2D MXenes have high electrical conductivity and are suitable for use as an interactive substrate to support active materials (MnO_2_,^[^ ^31^ ^]^ V_2_O_5_,^[^ ^32^ ^]^ VO(CH_2_O)_2_)^[^ ^33^ ^]^ in AZIBs cathodes, resulting in increased rate capability and cycle life. The high conductivity can ensure faster charge‐transfer kinetics, whereas the favorable surface functionalities enable the effective loading of active materials for improving the structural stability and cycling performance of the composite electrode materials.^[^ ^34^ ^]^ For example, MXene has been utilized as a conductive substrate for V‐based oxides such as H_2_V_3_O_8_. Due to its intrinsic low electrical conductivity and structural degradation caused by repetitive insertion/extraction of Zn ions during charge/discharge cycles, H_2_V_3_O_8_ exhibits poor rate performance and cycling stability. To overcome this issue, H_2_V_3_O_8_ nanowires (NW) were directly grown on the Ti_3_C_2_T*_x_* MXene sheets using a hydrothermal route.^[^ ^35^ ^]^ The Ti_3_C_2_T*_x_* not only supports the growth of H_2_V_3_O_8_ NW but also acts as a conductive substrate. The H_2_V_3_O_8_ NW/MXene composites realize a significantly large capacity of 365 mAh g^−1^ at 200 mA g^−1^ (306 mAh g^−1^ in pure H_2_V_3_O_8_). Furthermore, the utilization of Ti_3_C_2_T*_x_* MXene as a conductive substrate promotes the enhanced electrode kinetics of tris(aza)pentacene (TAP) by facilitating the diffusion of Zn^2+^ ions.^[^ ^36^ ^]^ Close electrical interactions between TAP and Ti_3_C_2_T*_x_* sustain the structural integrity of TAP/Ti_3_C_2_T*_x_* throughout multiple charge/discharge cycles. Consequently, the TAP/Ti_3_C_2_T*_x_* cathode delivers a remarkable capacity of 303 mAh g^−1^ at 40 mA g^−1^ in AZIBs, while maintaining an ultra‐long lifetime of over 10 000 cycles with an impressive capacity retention of 81.6%.

**Figure 3:**
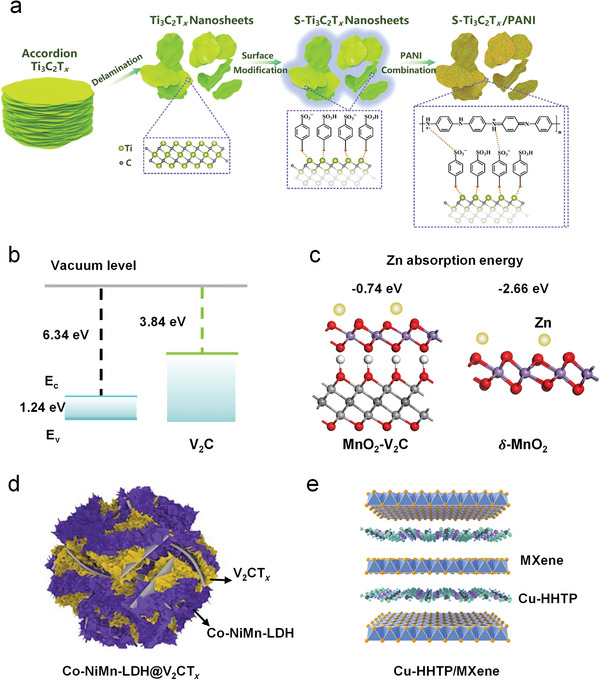
2D conductive substrate function. a) Schematic of the preparation of S‐Ti_3_C_2_T*_x_*/PANI. Reproduced with permission.^[^ ^27^ ^]^ Copyright 2021, American Chemical Society. b) Band edge positions of δ‐MnO_2_ and V_2_C, along with c) calculated absorption energies for Zn^2+^ ions on MnO_2_‐V_2_C and δ‐MnO_2_. Reproduced with permission.^[^ ^28^ ^]^ Copyright 2021, American Chemical Society. Schematic of the d) Co‐NiMn‐LDH@V_2_CT*_x_*. Reproduced with permission.^[^ ^29^ ^]^ Copyright 2022, Elsevier, and e) 2D CuHHTP/MX heterostructure. Reproduced with permission.^[^ ^30^ ^]^ Copyright 2023, Wiley‐VCH.

Grafting target termination onto the surface of Ti_3_C_2_T*_x_* MXene can further enhance the electronic characteristics of substrate. The charge‐carrier density and electrical conductivity of Ti_3_C_2_T*_x_* can be effectively promoted by precisely controlling the quantity and type of surface terminations. This can be achieved through techniques such as vacuum annealing and substitution reactions using molten inorganic salts, allowing for the regulation of surface terminations with elements like Cl, I, Br, S, NH. Thus, due to its improved electronic properties, the grafted Ti_3_C_2_T*_x_* also can be utilized as a conductive substrate to provide support for active materials in AZIBs cathodes. For example, polyaniline (PANI) is considered a highly promising polymer cathode material for AZIBs owing to its redox activity, but the electrochemical performance of PANI‐based electrodes is often hindered by its instability during repeated charge/discharge owing to deprotonation and swelling/shrinking. Here surface engineering involving the grafting of ‐SO_3_H groups on the Ti_3_C_2_T*_x_* (S‐Ti_3_C_2_T*_x_*) using a facile diazotization‐coupling reaction has been proven effective (**Figure** **3**a). To solve the deprotonation and swelling/shrinking difficulties of PANI during the electrochemical redox process, grafted MXenes with the capacity to operate as an interactive conductive substrate with adequate flexibility were utilized.^[^ ^27^ ^]^ The S‐Ti_3_C_2_T*_x_*/PANI cathode demonstrates excellent electrochemical performance and achieves a high discharge capacity of 262 mAh g^−1^ at 0.5 A g^−1^. Furthermore, it exhibits good rate capability, delivering a capacity of 160 mAh g^−1^ even at 15 A g^−1^. And the S‐Ti_3_C_2_T*_x_*/PANI cathode displays good cyclability, maintaining its performance over 5000 cycles with a coulombic efficiency of 100%.

In addition, V_2_CT*_x_* MXenes can be utilized as a conductive substrate for active materials to improve electrochemical performance. An in situ route to grow δ‐MnO_2_ over V_2_CT*_x_* and simultaneous intercalation of V_2_CT*_x_* was obtained by K^+^ (K‐V_2_C@MnO_2_). Here, the layered V_2_CT*_x_* substrate prevents MnO_2_ volume expansion and contraction, effectively delaying the irreversible dissolution of metal oxides and associated side reactions. The DFT calculations (Figure 3b) reveal the electron affinity of δ‐MnO_2_ and V_2_CT*_x_* to be 6.34 and 3.84 eV, respectively. While δ‐MnO_2_ acts as a semiconductor, V_2_CT*_x_* demonstrates metallic properties. Considering the band edge positions, electrons from V_2_CT*_x_* tend to transfer into the conduction band of MnO_2_, thereby enhancing the electronic conductivity of δ‐MnO_2_. Additionally, the presence of the V_2_CT*_x_* substrate affects the adsorption of Zn^2+^ ions on the surface of δ‐MnO_2_. Gibbs free energy calculations (Figure 3c) indicate a decrease in Zn^2+^ adsorption energy upon the introduction of the substrate. The results indicate that more Zn^2+^ might be desorbed from K‐V_2_C@MnO_2_ during desorption, which renew the electrochemically active surface area for subsequent adsorption, resulting in a high‐rate capacity. Thus, the K‐V_2_C@MnO_2_ shows a high capacity of 408.1 mAh g^−1^ at 300 mA g^−1^ and has good rate performance (87.7 mAh g^−1^ at 15 A g^−1^). Additionally, Han et al. employed a doping‐electrostatic synergic assembly route to synthesize the 2D Co‐doped NiMn‐Layered double hydroxide (LDH)/V_2_CT*_x_* (Figure 3d).^[^ ^29^ ^]^ The negatively charged 2D V_2_CT*_x_* acts as a conductive substrate, while the positively charged Co‐doped LDH provides rich electrochemical active sites and inhibits LDHs agglomeration during cycling. When it is tested as a cathode, the composite realizes a high capacity of 322.7 mAh g^−1^ at 200 mA g^−1^ after 100 cycles and superior rate performance (65.6 mAh g^−1^ at 5 A g^−1^). A heterostructure of 2D Cu‐HHTP active material and V_2_CT*_x_* was created to establish efficient charge‐transfer channels between Cu‐HHTP and MXene (Figure 3e).^[^ ^30^ ^]^ The presence of MXene helps to facilitate fast electronic transport, resulting a remarkable rate capability (173.1 mAh g^−1^ at 4 A g^−1^).

### 2.2 MXenes as a 3D Framework for Active Materials

**Figure 4:**
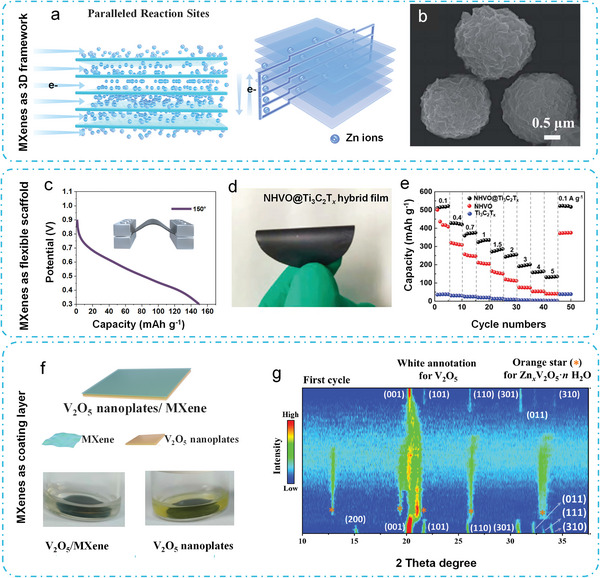
3D Framework, flexible scaffold and coating functions. a) Proposed Zn ion intercalation and electron flow (“parallel circuitry“) in the MnO*_x_* @Ti_3_C_2_T*_x_* multilayers. Reproduced with permission.^[^ ^37^ ^]^ Copyright 2019, Wiley‐VCH. b) SEM image of 3D Ti_3_C_2_T*_x_* @MnO_2_ microspheres. Reproduced with permission.^[^ ^38^ ^]^ Copyright 2020, Royal Society of Chemistry. c) Discharge curves of electrodes at bend angle of 150^○^. Reproduced with permission.^[^ ^39^ ^]^ Copyright 2021, Wiley‐VCH. d) Optical image of flexible NHVO@Ti_3_C_2_T*_x_* and (e) its rate performance. Reproduced with permission.^[^ ^40^ ^]^ Copyright 2021, Wiley‐VCH. f) Electrodes placed in a 3 M Zn(CF_3_SO_3_)_2_ aqueous solution for a period of 7 days and g) a contour plot of in situ XRD characterization was used to analyze the zinc storage behavior of a V_2_O_5_/MXene cathode. Reproduced with permission.^[^ ^32^ ^]^ Copyright 2021, American Chemical Society.

It has been demonstrated that a 3D interpenetrating network with various conductive pathways enhances charge‐transfer and ion diffusion rates. 2D lamella MXenes might be successfully turned into 3D MXenes utilizing structural modification and intrinsic conductivity. The MnO*_x_* functionalized MXene stacks (Ti_3_C_2_T*_x_*) with CNTs was prepared to form a 3D interpenetrating conductive structure.^[^ ^37^ ^]^ The composite serves its purpose by providing a parallel circuitry (**Figure** **4**a) for ions and electrons at the nano‐ and micro‐scale, which allows for a very high‐rate capacity. However, the MnO*_x_* @Ti_3_C_2_T*_x_* cathode only retains 50% of its capacity (88 mAh g^−1^) while the current density rises from 0.1 to 10 A g^−1^.

The 3D interpenetrating Ti_3_C_2_T*_x_* has also been integrated hydrothermally with ZnMn_2_O_4_ as the cathode for AZIBs.^[^ ^41^ ^]^ The high‐conductive multilayer Ti_3_C_2_T*_x_* scaffolds offer continuous conductive routes for effective electronic transport while simultaneously inhibiting the aggregation and dissolution of ZnMn_2_O_4_, hence delaying the irreversible structural deterioration and side reactions of ZnMn_2_O_4_ during cycles. The ZnMn_2_O_4_@Ti_3_C_2_T*_x_* cathode exhibits a reversible capacity of 172.6 mAh g^−1^ and long‐term cyclic stability (≈92.4% retention after 5000 cycles). Similarly, a 3D hydrated vanadium pentoxide/Ti_3_C_2_T*_x_* MXene composite using a one‐step hydrothermal method was reported.^[^ ^42^ ^]^ This composite shows a reversible capacity of 323 mAh g^−1^ at 100 mA g^−1^ and demonstrates exceptional rate capability that is attributed to its unique structure and high electrical conductivity. A 3D micro flower‐like Ti_3_C_2_T*_x_*‐MnO_2_ composite (Figure 4b) was prepared by gas‐phase spray drying method, in which the MnO_2_ nanoparticles are embedded by corrugated MXene nanosheets, realizing rapid ion/electron transfer capability and high structural stability.^[^ ^38^ ^]^ The 3D MXene@MnO_2_ micro flowers were used as an AZIB cathode and exhibits a reversible capacity of 301.2 mAh g^−1^ at 200 mA g^−1^ with a remarkable rate capability and cycling stability over 2000 cycles. A 3D bird nest‐like VO_2_/MXene composite also helps to mitigate the volume changes of the electrodes and enhance the kinetics of the active materials throughout cycling, leading to improved high‐rate performance, namely, 225.0 mAh g^−1^ after 4000 cycles at 30.0 A g^−1^.^[^ ^43^ ^]^ Multiple mechanisms govern MXene as a 3D framework supporting various zinc storage active materials. Its primary function is to stabilize the structure of the active materials during repeated cycles, to promote the transfer of electrons in 3D space, and to drive the rapid de/intercalation of Zn^2+^ in the host material.

### 2.3 MXenes as a Flexible Scaffold for Active Materials

MXenes flakes are easily transformable into flexible, additive‐free films with remarkable electrochemical properties and the potential for wearable, flexible electronics. These qualities make MXenes an appealing substrate for active loading materials, particularly for energy storage applications. MXenes are an excellent electron conductor and current collector in these composite‐based electrodes, promoting ion/electron transport throughout the cycling process. The incorporation of active materials limits the restacking of MXene‐flakes, hence conserving the active surface sites for effective charge storage properties. In addition, its adaptable and self‐supporting framework possesses the ability to buffer volume increase, provide extra active sites, and expedite ion transport. The flexible VO_2_/MXene hybrid films were fabricated by filtering a mixture of VO_2_ nanobelts and Ti_3_C_2_T*_x_*‐MXene.^[^ ^39^ ^]^ This resulted in the formation of a lamella‐like conductive network with strong mechanical properties and good bendability, where the MXene flakes act as robust, flexible, and conductive centers in combination with VO_2_ to facilitate fast ion‐kinetic transportation. Additionally, the electrode integration‐based technique employed eliminates the need for an extra binder and current collector, offering advantages in terms of increased energy and power density. Assembled as flexible quail‐solid‐state ZIBs, the prepared battery exhibits excellent mechanical adaptability, withstanding a large mechanical deformation angle of 150° without damage, while maintaining stable discharge capacity (Figure 4c).

Moreover, Ti_3_C_2_T*_x_* MXene could serve as a flexible scaffold for (NH_4_)_2_V_10_O_25_ · 8H_2_O (NHVO) to construct “rocking‐chair” AZIBs.^[^ ^40^ ^]^ The NHVO@Ti_3_C_2_T*_x_* flexible film has been prepared using a simple vacuum filtering of 1D ultrathin NHVO nanobelts with a high concentration of ultrathin Ti_3_C_2_T*_x_* nanosheets. The Ti_3_C_2_T*_x_* offers a flexible substrate for loading the NHVO nanobelts, which might have promoted the transport of ions/electrons over the whole electrode. The NHVO@Ti_3_C_2_T*_x_* hybrid film with engineered structures exhibits superior flexible cathode characteristics (Figure 4d) and delivers a remarkable capacity of 514.7 mAh g^−1^ (Figure 4e), enduring 6000 cycles with 84.2% retention at 5.0 A g^−1^.

### 2.4 MXene as Coating Layer of Active Materials

With their multi‐electron redox reactions and wide ion transport channels, the most widely investigated vanadium oxide‐based materials are good candidates for AZIB cathodes. However, the storage performance of vanadium oxide cathodes for divalent Zn ions is limited due to several inherent electrochemical constraints. Vanadium tends to dissolve in acidic or neutral electrolytes, which results in structural degradation and electrolyte contamination. Another challenge associated with layered vanadium oxides is their subpar rate capability, which can be attributed to both the intrinsically low conductivity and the strong electrostatic repulsion within the layers. To address these challenges, our group investigated the use of Ti_3_C_2_T*_x_* as a coating layer on V_2_O_5_ nanoplates.^[^ ^32^ ^]^ This coating layer effectively suppresses vanadium dissolution, as observed in the progressive yellowing of the electrolyte impregnated with V_2_O_5_ nanoplates, while the color change is significantly mitigated in V_2_O_5_/MXene (Figure 4f). Additionally, in situ XRD results (Figure 4g) demonstrate that the MXene layer facilitates reversible co‐intercalation/deintercalation of water molecules and Zn^2+^ in the V_2_O_5_/MXene hybrid. Consequently, an enhanced electrochemical kinetics can be achieved, leading to a high‐rate performance.

### 2.5 MXenes Derivatives as Active Materials

The strong conductivity and rich surface chemistry of the 2D/3D MXenes generate multiple active sites and organized nanochannels which promote charge transfer and intercalation/deintercalation of Zn^2+^ ions in active materials. Nevertheless, the most widely studied Ti_3_C_2_(OF) has been reported to produce low redox activity with a capacity of only 51.7 mAh g^−1^ at a current density of 500 mA g^−1^ in AZIBs.^[^ ^44^ ^]^ Presently, modifying the surface terminals of MXenes is a promising strategy to improve their electrochemical activity in AZIBs.

**Figure 5:**
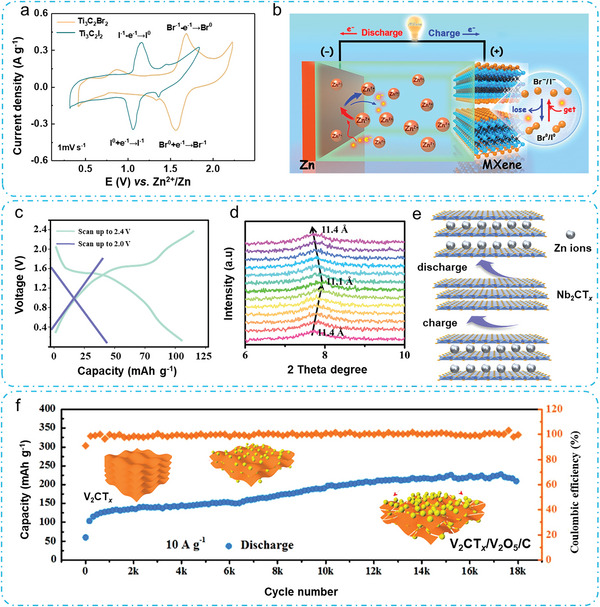
MXene as active materials or precursor in AZIBs. a) CV curves of Ti_3_C_2_Br_2_ and Ti_3_C_2_I_2_ at a scan rate of 1 mV s^−1^, and b) schematic illustration of a Zn‐Ti_3_C_2_‐Br/‐I cell. Reproduced with permission.^[^ ^44^ ^]^ Copyright 2021, American Chemical Society. c) GCD curves of Nb_2_CT*_x_* MXene recorded at 1 A g^−1^ up to 2.4 V, d) in situ XRD patterns of (002) plane at different discharge/charge cycles and e) schematic diagram of storage mechanism in Nb_2_CT*_x_*. Reproduced with permission.^[^ ^45^ ^]^ Copyright 2021, Elsevier. f) Prolonged cycling behavior of V_2_CT*_x_*‐based ZHIB at 10 A g^−1^, and diagram (inset) of phase transition product V_2_CT*_x_* /V_2_O_5_/C. Reproduced with permission.^[^ ^23^ ^]^ Copyright 2020, American Chemical Society.

MXenes are denoted by the formula M_n+1_X_n_T*_x_*, where T*_x_* represents the surface terminal group that depends on the etchant type and synthesis process. In general, HF or LiF/HCl etchants create ‐OH, ‐O, and ‐F‐based functionalities, whereas the Lewis‐acidic‐melt etching method produces ‐Cl, ‐Br, and ‐I. The composition and arrangement of these surface terminals significantly impact the physical and chemical properties of MXenes, including their electrochemical performance in aqueous zinc storage. To analyze the impact of various surface terminals, Ti_3_C_2_ are prepared using the Lewis‐acidic‐melt etching method.^[^ ^44^ ^]^ These MXenes include individual ‐Cl, ‐Br, and ‐I terminals, dual ‐ClBr, ‐ClI, and ‐BrI terminals, as well as trinary ‐ClBrI terminals. The discharge behavior of individual‐halogen‐terminated MXenes, specifically Ti_3_C_2_Br_2_ and Ti_3_C_2_I_2_, is different from conventional MXenes prepared by HF etching (referred to as Ti_3_C_2_(OF)). Ti_3_C_2_Br_2_ exhibits a discharge plateau at ≈1.6 V, while Ti_3_C_2_I_2_ displays a plateau at ≈1.1 V. Their specific capacities are 97.6 and 135 mAh g^−1^. Correspondingly, the CV curves (**Figure** **5**a) display characteristic redox peaks for both Ti_3_C_2_Br_2_ and Ti_3_C_2_I_2_. The peaks at 1.55/1.65 V are attributed to the reversible conversion of Br^−^/Br° for Ti_3_C_2_Br_2_, while the peaks at 1.05/1.15 V are assigned to the reversible conversion of I^−^/I° for Ti_3_C_2_I_2_, both referenced to the Zn/Zn^2+^ redox couple. Hence, the energy conversion reaction of Ti_3_C_2_Br_2_//Zn or Ti_3_C_2_I_2_//Zn cell (as illustrated in Figure 5b) is as follows.

(1) $$\mathrm{Cathode}:\;2Br^{-}-2e^{-}↔2Br^{0}\;\left(\;E_{0}=\;1.065\;\mathrm{vs}.\;\mathrm{SHE}\right)$$

(2) $$\mathrm{Cathode}:\;2I^{-}-2e^{-}↔2I^{0}\;\left(\;E_{0}=\;0.536\;\mathrm{vs}.\;\mathrm{SHE}\right)$$

(3) $$\mathrm{Anode}:\;Zn^{2+}+2e^{-}↔Zn^{0}\;\left(\;E_{0}=\;-0.763\;\mathrm{vs}.\;\mathrm{SHE}\right)$$

Furthermore, the MXenes with multiple halogen termination, specifically Ti_3_C_2_(BrI) and Ti_3_C_2_(ClBrI), show pair discharge plateaus and achieve a capacity of 117.2 and 107.4 mAh g^−1^, respectively. However, Ti_3_C_2_Cl_2_ and Ti_3_C_2_(OF) exhibit lower capacities of 46.5 and 51.7 mAh g^−1^, respectively, in the absence of distinct discharge plateau. The exceptional electrochemical properties observed in the halogenated MXenes are assigned to the reversible redox behavior of the ‐Br and ‐I groups located on the outermost Ti layers within the Ti_3_C_2_ structure.

Expanding the voltage is an effective approach to achieving high discharge capacity and energy density of Nb_2_CT*_x_*. The multilayer Nb_2_CT*_x_* exhibits a voltage plateau of 1.55 V with a battery‐like behavior (Figure 5c) in the extended voltage window of 2.4 V in a high‐concentration (21 M) electrolyte containing LiTFSI+1 M Zn (OTf)_2_.^[^ ^45^ ^]^ When the voltage is below 2.0 V, the device exhibits typical capacitive behavior. However, beyond 2.4 V the discharge capacity and energy density of Nb_2_CT*_x_* show significant improvement. For example, at a discharge current density of 500 mA g^−1^, the capacity is more than 2.5 times that achieved at 2.0 V, corresponding to an energy density 3 times that obtained at 2.0 V. The storage mechanism of Nb_2_CT*_x_* has been explained based on in situ XRD measurements which unravels the phase composition evolution as the voltage reaches up to 2.4 V (Figure 5d). It is shown that structure change is reversible, which is consistent with intercalation/extraction behavior of zinc ions (Figure 5e). Hence, the insertion/extraction of zinc ions are mostly responsible for the observed battery behavior, and the possible reactions of the Nb_2_CT*_x_*/Zn cell can be described as follows:

(4) $$\mathrm{Cathode}:Nb_{2}CT_{x}+\mathrm{yZ}n^{2+}+2ye^{-}↔2Nb_{2}Zn_{y}CT_{x}$$

(5) $$\mathrm{Anode}:\;yZn-2ye^{-}↔yZn^{2+}$$

It is important that the electrode materials exhibiting battery behavior and high output voltage can contribute significantly to the overall capacity. Nevertheless, a capacitive characteristic is common in MXene‐based electrodes for aqueous based supercapacitors. In addition, the continuous oxidation of MXenes under high voltages limits the potential window below 1.5 V, resulting in an energy storge density less than 100 Wh kg^−1^. Such a predominantly capacitive or pseudocapacitive behavior also exists in non‐aqueous secondary batteries including Li, Na, K, and Al ions batteries. This leads to distorted rectangular cyclic voltammetry profiles and a linear charge–discharge feature. Consequently, the voltage output of capacitive MXene electrodes diminishes rapidly compared to battery‐type systems that exhibit distinct and stable discharge regions.

From the above analysis, we may conclude that, among the MXenes‐based cathode materials for AZIBs, Ti_3_C_2_(OF)‐MXene demonstrates poor electrochemical activity. In contrast, halogenated MXenes exhibit redox peaks originating specifically from the ‐Br and ‐I terminations. Additionally, Nb_2_CT_x_ displays battery behavior due to zinc ion intercalation and deintercalation at high voltage of 2.4 V. To elucidate the high electrochemical activity of MXenes electrode materials, we will discuss V_2_CT*_x_* as an active material precursor featuring redox peaks attributed to VO_x_ in the next section.

### 2.6 MXenes as Precursor of Active Materials

The V_2_CT*_x_* MXene, which has V atoms with inherently modest valence states of +2 and +3, does not engage in multielectron redox processes during charge/discharge cycles for AZIBs. Enhancing the valence state of the V atoms in V_2_CT*_x_* makes it feasible to increase the redox activity of the V_2_CT*_x_*‐based AZIBs cathodes. Current research indicates that the V_2_CT*_x_* as a precursor is oxidized or weakly oxidized under different direct or indirect oxidation treatments to function as active material (VO*_x_*, x>3). In addition, the surface selenization strategy for V_2_CT*_x_* can also realize the conversion of MXene surface metal atoms into transition metal selenides (TMSes, such as VSe_2_) while preserving the inner layer of MXene to ensure high capacity and good structural stability.^[^ ^46^ ^]^ It is clear that vanadium oxides or TMSes, which use the surface atoms and the peculiar 2D structure of MXene, are responsible for exhibiting electrochemical activity in these cathodes, and thus, MXene works as a precursor to active materials.

#### 2.6.1 Indirect Oxidation Treatment of V_2_CT*_x_*

A zinc hybrid‐ion battery (ZHIB) with a 2D multilayer V_2_CT*_x_* cathode active material produced by HF etching displays a significant capacity gain after more than 18 000 cycles (Figure 5f).^[^ ^23^ ^]^ The growing capacity observed can be attributed to the progressive phase change of the V_2_CT*_x_* cathode into V_2_O_5_. This transition leads to a continuous contribution to the capacity by means of a distinct reaction (shown in the inset of Figure 5f). The original cathode material and the secondary phase change product work in tandem to contribute to the overall capacity. As a result, even after 1600 cycles at a very high current of 10 A g^−1^, the electrode retains a capacity of 202 mAh g^−1^, indicating the sustained performance of the battery over time. This is an enticing revelation for batteries with a long lifespan, where the valence state of V atoms promotes the electrochemical activity. In another study, partial phase transition from V_2_CT*_x_* to V_2_O*_x_* (V_2_O*_x_* @V_2_CT*_x_*) is observed which might occur during a high‐temperature etching method (in situ HF method with NaF and HCl solution at 90°C) and also during electrochemical activation process.^[^ ^47^ ^]^ This phase transition, according to authors, endows the V_2_O*_x_* @V_2_CT*_x_* cathode in AZIBs with high discharge capacity of over 300 mAh g^−1^ at 0.05 A g^−1^ and an extend cyclic stability with over 80% capacity retention after 200 cycles.

While the above approaches are efficient, the traditional HF‐synthesis route to preparing V_2_CT*_x_* is not environmentally friendly. Interestingly the MAX phase, i.e., V_2_AlC, may be immediately exfoliated inside the battery (in situ) by selecting a particular F‐rich electrolyte.^[^ ^48^ ^]^ This one‐step process, in which all reactions occur within the cell, prevents hazard to the environment due to HF. The overall cell reaction process was divided into three phases: exfoliation, electrode oxidation, and redox activity of V_2_O_5_. Despite the regular phase evolution of electrodes, the battery shows continuous increase in capacity within first 2000 cycles.

Additionally, indirect oxidation treatment of V_2_CT*_x_* can be accompanied by cooperative intercalation ions in V_2_CT*_x_*. Since AZIB cathodes operate on the principle of ion insertion/extraction, it is crucial to have large lattice channels that facilitate efficient and reversible accumulation of Zn^2+^ ions, thereby enhancing energy storage capabilities. An effective strategy is introducing second cations, such as Li^+^, Na^+^, Zn^2+^, Mn^2+^, and Al^3+^, as the “pillar” into these bilayers and expanding interlayer spacing. The pre‐intercalation of cations in this manner serves two important purposes. First, it effectively mitigates the high charge density of multivalent ions, providing a buffering effect. Second, it reduces the energy required for de‐solvation of carrier ions through simple hydration, thereby lowering the activation energy associated with electron transfer at the interface‐region between the electrode and electrolyte. As a result, this pre‐intercalation process enhances the overall efficiency of charge transfer in the system. The heterostructure composite of Mn^2+^‐intercalated hydrated vanadate and remnant V_2_CT*_x_* skeleton (MVO@VC) was fabricated for AZIBs cathodes via in situ hydrothermal derivatization.^[^ ^49^ ^]^ By combining intercalated Mn^2+^ with the intrinsic conductive framework of V_2_CT*_x_*, the diffusion kinetics of Zn^2+^ are greatly improved, along with the enhanced structural stability of hydrated vanadate. As a result, the MVO@VC electrode exhibits exceptional rate capacity, delivering 235.5 mAh g^−1^ at an elevated current density of 22 A g^−1^, and preserving 58.3% of its initial value when cycles at a slower current density of 0.5 A g^−1^. This demonstrates the superior performance of the MVO@VC electrode in terms of both high‐rate capability and capacity durability. In addition, the V_2_CT*_x_*‐Zn*_x_* V_2_O_5_∙nH_2_O (VC‐ZVO) was sourced from pre‐alkaline treated V_2_CT*_x_* by simultaneous Zn^2+^ intercalation and oxidation.^[^ ^50^ ^]^ The pre‐intercalation of Zn^2+^ ions and the formation of interfaces between ZVO and residual V_2_CT*_x_* contribute to enhanced structural stability and reduced electrostatic interactions. The prepared VC‐ZVO cathode presents a high‐rate performance (223.9 mAh g^−1^ at 10 A g^−1^) and durable cycling capability (≈96.4% capacity persistence over 8000 cycles).

#### 2.6.2 Direct Oxidation Treatment of V_2_CT*_x_*

*An* in situ electrochemical activation tactic was proposed to directly oxidize the V_2_CT*_x_* cathode via initial charging at a specific potential (1.4, 1.8, and 2.0 V), leading to the creation of a secondary phase, specifically transition metal oxides, on the surface of V_2_CT*_x_*.^[^ ^51^ ^]^ This direct electrochemical pre‐oxidation of V_2_CT*_x_* is categorized as a weak oxidation treatment, which leads to a rise in the valence state of V from V^2+^/V^3+^ to V^4+^/V^5+^ while maintaining the integrity of the inner conductive V‐C‐V 2D framework.^[^ ^51^ ^]^ The X‐ray absorption fine structure (XAFS) analysis indicates an elevated oxidation state of V atoms close to VO_2_ and V_2_O_5_ after charge activation of 1.8‐V_2_CT*_x_*. In addition, XRD measurements demonstrate that the peaks at (002), (103) and (112) of 1.8‐V_2_CT*_x_* are still present, indicating that the conductive V‐C‐V 2D structure has been preserved despite considerable oxidation. Consequently, the VO_x_/V_2_CT*_x_* heterostructure includes the external layer of highly oxidized V and the internal conductive V‐C‐V 2D architecture, enabling the activated V_2_CT*_x_* cathode to undergo multiple electron redox processes, realizing a high capacity of 423.5 mAh g^−1^ at 1 A g^−1^ and a rapid Zn^2+^ ions storage capability of 358 mAh g^−1^ at 30 A g^−1^.

Vanadium pentoxide is the active material of the AZIBs cathode, which may be produced by direct oxidation of V_2_CT*_x_* utilizing stronger oxidation techniques. For example, a morphology‐controlled growth of V_2_O_5_ by oxidizing V_2_CT*_x_* was reported via a simple hydrothermal process under various temperatures (such as 160, 180 and 200 ^○^C) and used as a cathode for AZIBs.^[^ ^52^ ^]^ The controlled growth of V_2_O_5_ on the surface of V_2_CT*_x_*, which possesses a low interfacial resistance, prevents volume expansion and enhances ions diffusion. The V‐based cathode oxidized at 180 ^○^C delivers a significant capacity of 397 mAh g^−1^ at 0.5 A g^−1^, outstanding rate capacity and sustained cycling stability.

A one‐step annealing procedure may also be adapted to synthesize morphology‐controllable micron‐sized nanoporous V_2_O_5_ arrays from V_2_CT*_x_*.^[^ ^53^ ^]^ The crystallinity, microstructure, and electrochemical performance of V_2_O_5_ were examined under various annealing conditions (0.1 and 1 °C min^−1^ heating rates and in the temperature range from 250 to 650 °C). The rationally designed V_2_CT*_x_*‐350‐0.1, which was obtained by thermally treating V_2_CT*_x_* in the air, demonstrates a unique porous architecture and 2D structure. These characteristics render high ion accessibility, structural stability, and rapid charge transport. When employed as a cathode material for gel zinc‐ion batteries, the V_2_CT*_x_*‐350‐0.1 exhibits reasonably good performance.

#### 2.6.3 Surface Selenization Treatment of V_2_CT*_x_*

2D transition metal selenides (TMSes) have been considered promising hosts for Zn^2+^ due to their layered structures, large interlayer spacing, and metallic conductivity (e.g., 1000 S cm^−1^ for VSe_2_). However, TMSes face challenges related to strong layer restacking, resulting in inferior rate capacity and cyclability. To address this issue, a surface selenization strategy has been proposed, specifically for constructing TMSes on MXenes. In this surface selenization procedure, the metal atoms on the surface of MXenes serve as the metal sources for the formation of TMS nanoplates (TMSe) in situ. Additionally, the underlying layers of MXenes serve as a substrate, preventing the self‐stacking of TMSe and immobilizing the TMSe nanoplates. This surface selenization approach can be applied to various MXenes. For instance, VSe_2_@V_2_CT*_x_* nanohybrids are obtained by surface selenization of V_2_CT*_x_* and investigated as cathode materials for AZIBs.^[^ ^46^ ^]^ The VSe_2_@V_2_CT*_x_* nanohybrids exhibit improved Zn^2+^ diffusion kinetics, leading to good rate capability and cycling stability. Specifically, they demonstrate capacities of 231.3 and 158.1 mA h g^−1^ at 0.5 and 2.0 A g^−1^, respectively, after 100 and 600 cycles. The V_2_CT*_x_* MXenes, with their remarkable hydrophilicity, excellent metallic conductivity, and well‐defined layered structure, benefits the Zn^2+^ diffusion. And VSe_2_, with noteworthy capacity and stable structural robustness, contributes to stable Zn^2+^ storage. Morphologically, V_2_CT*_x_* provides a flexible substrate for loading VSe_2_ nanoplates, preventing their stacking. When MXenes are used as precursors, this type of electrode material demonstrates high electrochemical activity and outstanding performance, making it worthy of further exploration.

## 3 MXenes in the AZIBs Anodes

Zinc metal has a high theoretical capacity, volumetric capacity (5855 mAh cm^−3^), and significant redox potential (−0.763 V vs. SHE).^[^ ^54^ ^]^ Additionally, Zn metal has the advantages of a low price (2 USD kg^−1^), safety, and environmental friendliness, as well as a plentiful supply of resources.^[^ ^55^ ^]^ Despite the multiple benefits, using Zn metal anodes is challenging due to critical issues such as dendrite development, dead zinc, parasitic reactions, and prolonged battery stability. Major initiatives are underway to extend the life of the relatively thin Zn metal anodes.^[^ ^56^ ^]^ The common strategies involve host design,^[^ ^57^ ^]^ electrode surface modification,^[^ ^58^ ^]^ electrolyte regulation,^[^ ^59^ ^]^ and alloyed treatment^[^ ^60^ ^]^ to enhance the redistribution of ions and electrons on the surface of the Zn anode. As a 2D material with several surface functional groups and the potential to be fashioned into a wide range of macroscopic forms, MXene shows great promise for addressing issues with metal anodes. Various MXene‐assisted strategies for achieving stable and dendrite‐free zinc metal anodes have been explored, ranging from designing an MXene host, modifying the surface of zinc, to coating separators or electrolytes with MXene. In this section, we present a summary of recent progress on improvement strategies and their electrochemical performance.

### 3.1 MXene‐Based Host Design

The “hostless” nature and susceptibility to dendrites result in unlimited volume variations of a zinc anode. These challenges can be addressed by designing diverse hosts for zinc metal anodes. Here the following merits make MXene an ideal choice for a host.^[^ ^61^ ^]^ The high metallic conductivity of MXenes (15 000 S cm^−1^) and ion diffusion coefficient facilitate fast electrochemical kinetics in the plating/stripping of Zn. Ti_3_C_2_T*_x_* colloidal solution can be easily fabricated into flexible and freestanding Ti_3_C_2_T*_x_* paper, allowing it to be used as a current collector without any additives. The abundant surface functional groups of MXene are tailorable for optimal Zn^2+^ adsorption and diffusion. In addition, MXene can be linked with zincophilic seeds to generate a new host, which will assure a dendrite‐free zinc surface. Finally, MXene may be readily processed into 3D scaffolds to improve the zinc ion transport flux without altering the original electron transport properties.

#### 3.1.1 2D MXene Host

**Figure 6:**
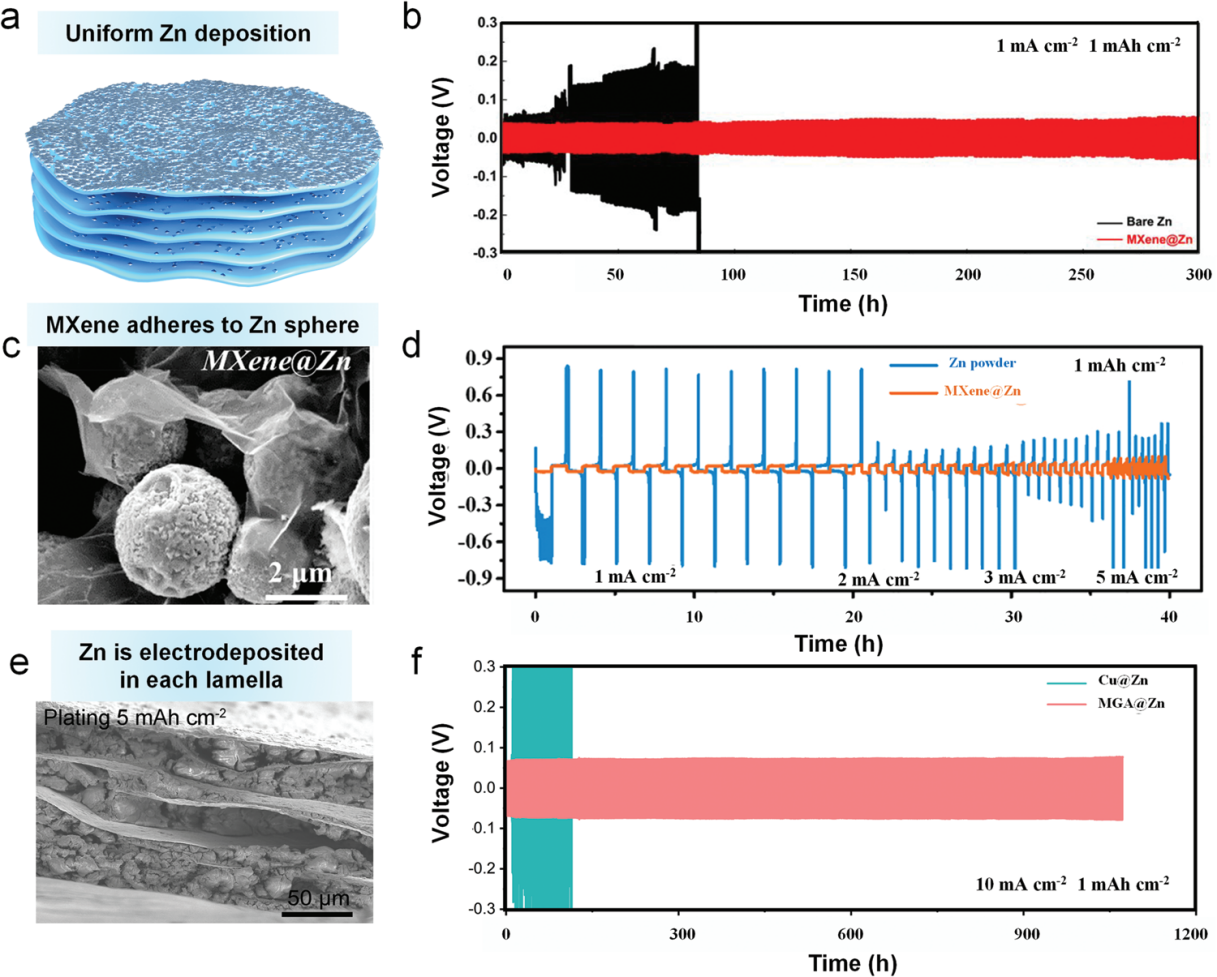
MXenes as the host of zinc anode. a) Schematic illustration of Zn deposition on Ti_3_C_2_T*_x_* MXene@Zn, and b) voltage profiles of Zn plating/stripping on uncoated Zn anode and Ti_3_C_2_T*_x_* MXene@Zn anode. Reproduced with permission.^[^ ^62^ ^]^ Copyright 2019, American Chemical Society. c) SEM image of MXene@Zn powder composites and d) voltage profiles of Zn stripping/plating at the different current densities with a constant capacity of uncoated Zn powder and MXene@Zn symmetric cells. Reproduced with permission.^[^ ^63^ ^]^ Copyright 2021, American Chemical Society. e) SEM image of MXene/graphene aerogel@Zn and f) cycling performance of symmetrical cells employing Cu@Zn and MXene/graphene aerogel@Zn electrodes. Reproduced with permission.^[^ ^65^ ^]^ Copyright 2022, Wiley‐VCH.

In situ electrodepositing Zn fabricated the Ti_3_C_2_T*_x_* MXene@Zn paper onto 2D MXene host in ZnSO_4_ electrolyte (**Figure** **6**a).^[^ ^62^ ^]^ The Ti_3_C_2_T*_x_* MXene@Zn anode can suppress the emergence of Zn dendrites as a consequence of its layered structure, outstanding metal conduction, hydrophilicity, and pliability. Ti_3_C_2_T*_x_* MXene@Zn anode demonstrates consistent cycling performance with a low polarization overpotential (≈75 mV) after 150 cycles. (Figure 6b). In addition, MXene@Zn anodes can be prepared by a simple solution‐mixing process where MXene is coated with zinc powder (Figure 6c).^[^ ^63^ ^]^ Zinc powder is an inexpensive and readily available commercial product. However, Zn powder exhibits quickly degrading overpotentials (not exceeding 10 h at 1 mAh cm^−2^), even worse than zinc foil (not exceeding 150 h at 1 mAh cm^−2^).^[^ ^63^ ^]^ This degradation may be attributed to the lack of poor ion distribution and electronic conduction. Due to the exceptional hydrophilicity and conductivity of flexible 2D Ti_3_C_2_T*_x_* MXene flakes, it will induce the redistribution of electrons/ions and reversibility of zinc powder during stripping/plating. Consequently, the fluctuating overpotential of the MXene@Zn anode is negligible, and the polarization voltage is less than 100 mV (Figure 6d).

#### 3.1.2 Surface‐tuned MXene Host

Selective etching procedures using fluoride‐containing acidic solutions have been commonly employed to create MXenes, with ‐O, ‐OH, ‐F, and ‐Cl functional groups as common terminations. However, MXenes produced through such methods often exhibit non‐uniform nucleation sites for the Zn anode and uneven current distribution. To address this issue, Yi et al. developed LF‐Ti_3_C_2_T*_x_* MXene with a low‐content of F functional group by subjecting Ti_3_C_2_T*_x_* to high‐temperature treatment in an inert atmosphere.^[^ ^64^ ^]^ This process results in the formation of a robust skeleton for the Zn anode. The LF‐Ti_3_C_2_T*_x_* MXene enables even plating of Zn ions, leading to the horizontal growth of Zn on its surface. The lamellar structure and high electrical conductivity of the Ti_3_C_2_T*_x_* scaffold, with a low F functional group content, provides rapid ionic transport pathways, uniform current distribution, and uniform nucleation sites for Zn^2+^.^[^ ^62^ ^]^ Accordingly, the LF‐Ti_3_C_2_T*_x_*‐based Zn anode exhibits a minimal voltage hysteresis of 63 mV and an extended lifespan of over 280 h. These findings highlight the potential of LF‐Ti_3_C_2_T*_x_* MXene as a 2D material for high‐performance Zn anodes.

In addition, a series of MXenes was synthesized using a molten salt method with identical halogen functional groups (‐Cl, ‐Br, ‐I) as an artificially generated coating that induces uniform Zn plating.^[^ ^66^ ^]^ The optimal redistribution of zinc ions stems from coordinated heterogeneous boundary rebuilding and controlled ion tiling by halogen terminated surface. Zn^2+^ ions are led to nucleate evenly on the dominant (000l) crystal plane of the MXene matrix and grown planarly by the synergism of strong lattice agreement (90%) between the MXenes and Zn and the precise halogen‐functionalization control. In the case of termination regulation, Cl termination shows superior efficacy for Zn ion control than O/F, Br, and I because of its modest adsorption and diffusion coefficients for Zn^2+^ ions. The Ti_3_C_2_Cl_2_‐Zn anode demonstrates good cyclability, lasting over 12 times longer (840 h at 2 mA cm^−2^//1 mAh cm^−2^) compared to the bare Zn anode. Moreover, when paired with a Ti_3_C_2_I_2_ cathode and Ti_3_C_2_Cl_2_‐Zn anode, the cell exhibits remarkable stability more than 9000 cycles at 3 A g^−1^.

#### 3.1.3 MXene with Zincophilic Seeds Host

The intrinsic functional groups (‐F, ‐Cl) in MXene for zinc anode can work as nucleation sites to improve zincophilicity and control zinc deposition. However, the degree of zincophilicity is not sufficient dependent on these functional groups. Combining MXene with zincophilic seeds is an effective strategy for enhancing zincophilicity. Generally, the alloying can provide sites that promote zinc nucleation, minimize nucleation overpotential, as well as boost deposition reliability. The antimony (Sb) modified Ti_3_C_2_T*_x_* MXene host was fabricated by electrodeposition and galvanic replacement approaches for dendrite‐free Zn anodes.^[^ ^67^ ^]^ When Sb is evenly grown on MXene paper, it is capable of reversibly alloying with zinc to create ZnSb intermetallic compound and acting as zincophilic nucleation sites. The MXene@Sb anode can produce homogeneous Zn deposition and inhibit side reactions owing to the zincophilic antimony seeds and MXene host, realizing a long cycling life of up to 1000 h.

#### 3.1.4 3D MXene Host

MXene has significant restacking difficulties throughout the assembly process, which increases interface resistance and renders uniform zinc deposition ineffective. Therefore, achieving efficient dendritic suppression at greater discharge levels is challenging. In order to improve zinc ion mobility and maintain battery stability, single MXene nanosheets can be constructed as linked 3D macroscopic frameworks. A new 3D MXene/RGO hybrid hydrogel (MGA) as a zincophilic host for Zn wrapping was prepared by electrodeposition method (Figure 6e).^[^ ^65^ ^]^ The composite zinc metal anode harvests a long cycle life (>1000 cycles) at 10 mA cm^−2^ in an asymmetric cell (Figure 6f). A quasi‐solid‐state pouch cell with the MGA@Zn anode and LiMn_2_O_4_ (LMO) cathode demonstrates improved mechanical flexibility and negligible capacity degradation under different folding times.

Moreover, a heterogeneous protective layer comprising of S‐doped 3D MXene frameworks (S/MX) and ion‐transfer ZnS was developed for Zn metal anodes.^[^ ^68^ ^]^ This approach involves an in situ vapour‐solid route where S incorporation and formation of ZnS occurred concurrently. The S‐doped 3D MXene acts as an effective inhibitor of Zn dendrite formation by decreasing the polarization of Zn electrodeposition, mitigating volume fluctuations, and acting as a physical obstruction. The ion‐transfer ZnS layer plays a crucial role in facilitating the homogeneous distribution and transfers of Zn^2+^ while preventing side reactions. The effectiveness of the protective layer was evaluated by comparing the morphological evolution of Zn deposition on unmodified Zn and the S/MX@ZnS@Zn‐350 anode. In the case of the unmodified Zn, Zn dendrites expands into large, porous structures with a plating capacity of 5 mAh cm^−2^. However, the S/MX@ZnS@Zn‐350 anode maintains a relatively smooth surface even after reaching a plating capacity of 5 mAh cm^−2^, and exhibits good cycling stability over 1600 h at 0.5 mA cm^−2^. This innovative design of the heterogeneous protective layer combining S‐doped 3D MXene host and ion‐transfer ZnS holds great promise for enhancing the performance and durability of Zn metal anodes.

### 3.2 Modifying Zinc Surface with MXene

The in situ generated solid electrolyte interface (SEI) in lithium metal batteries with organic electrolytes protects the lithium anode from excessive electrolyte degradation.^[^ ^69^ ^]^ However, the competitive H_2_ evolution mechanism inhibits the creation of a continuous SEI on the Zn metal anode surface. Thus, fresh surface of the Zn anode will be progressively passivated by the inactive byproducts of H_2_ development, leading to an uneven charge distribution over time. Such destructive irreparable changes will spread at the Zn‐electrolyte interface, causing dendrite formation and a dramatic reduction in cycle performance with time. This section discusses the application of MXene‐based protective covering to stabilize zinc anode and suppress the formation of zinc dendrites.

**Figure 7:**
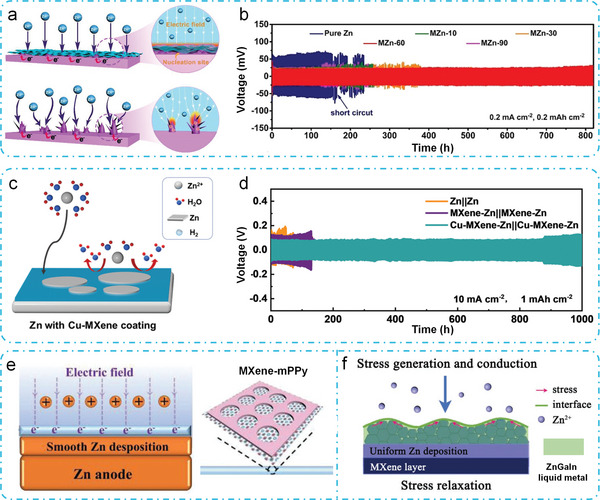
MXenes as modification layer of zinc anode. a) The illustration shows two parts: the upper part demonstrates the synchronous reduction and assembly of MXene layer on Zn foil, while the next part illustrates the plating behavior of pure Zn without any protective layer. b) Extended cycling performance of symmetric cells with pure Zn and MZn‐x (X = 10, 30, 60, 90). Reproduced with permission.^[^ ^70^ ^]^ Copyright 2021, Wiley‐VCH. c) Inhibition of side reactions of Cu‐MXene hydrophobic coating on Zn, and d) Zn striping/plating cycling stability of three types of symmetric cells. Schematic of Zn plating on e) MXene‐mPPy (Reproduced with permission.^[^ ^71^ ^]^ Copyright 2022, Wiley‐VCH.) and f) ZnGaIn//MXene (Reproduced with permission.^[^ ^72^ ^]^ Copyright 2022, Wiley‐VCH.) coated Zn anode.

An in situ, spontaneously reducing and assembling approach was employed to fabricate a uniform MXene thin layer on the surface of the Zn anode.^[^ ^70^ ^]^ Specifically, Zn foil is put on the MXene dispersion solution and the MXene sheets self‐assemble on Zn foil in a charge transfer process when the static repulsion between the MXene sheets is less than the bonding interaction between MXene and Zn surface. After drying, the MXene layer adheres to the Zn foil. The existence of the MXene layer on the Zn anode lowers the energy barrier for the nucleation of Zn and enhances a more homogeneous distribution of the electric field. This is attributed to the favorable charge redistribution effect provided by the MXene layer, in contrast to a pristine Zn anode (**Figure** **7**a). As a result, the MXene‐integrated Zn anode shows reduced voltage hysteresis (47 mV for 800 h) and good cycle stability with dendrite‐free characteristics (Figure 7b). The cell with optimized MXene decorated Zn anodes, and MnO_2_ cathode delivers good cycling stability, maintaining 81% of its initial capacity after 500 cycles. Afterwards, a Cu‐modified Ti_3_C_2_Cl_2_ MXene as a protective coating for Zn anodes was reported.^[^ ^73^ ^]^ Unlike other MXenes with ‐O, ‐OH, and F terminations, this MXene has only ‐Cl terminations, making it suitable for Zn^2+^ adsorption and diffusion. The addition of Cu provides nucleation sites and promotes homogeneous Zn^2+^ flux, which suppresses the growth of Zn dendrites (Figure 7c). The Cu‐MXene coating isolates the Zn electrode from the aqueous electrolyte, resulting in stable cycling, high reversibility, and excellent electrochemical kinetics. The coated Zn anode exhibits stable cycling for 1000 h at 10 mA cm^−2^, 1 mAh cm^−2^ in a symmetric cell (Figure 7d).

MXene‐based composites can help protect the zinc metal anode. The MXene‐mPPy was deposited on the surface of Zn foil by spray coating (Figure 7e).^[^ ^71^ ^]^ The exceptional and stable charge capacity of 149 F g^−1^ is achieved by the MXene‐mPPy layers, attributed to their unique sandwich structure and mesoporous PPy. This capacity enhancement is facilitated by the accumulation of charge and the homogeneous dispersion of electric field and ion flux. Consequently, both the Zn/Zn symmetric cell and the MnO_2_//Zn full cell, assembled with MXene‐mPPy/Zn anode, demonstrates outstanding rate capability and prolonged stability. Moreover, an adaptable protective layer based on self‐assembled MXene nanosheets and chitosan was applied by blade‐casting onto the zinc metal.^[^ ^74^ ^]^ In the case of artificial MXene/chitosan film, the Zn^2+^ concentration gradient near the anode is reduced by the strong coordination of amine groups provide by chitosan. It is claimed that the Zn dendrite growth is prevented because, according to the paper, the interconnected MXene component can help ioniz dendritic Zn and homogenize the electric field. The MXene/chitosan coated Zn anode exhibits a reversible plating/stripping life over 2100 h at 1.0 mA cm^−2^. Furthermore, to alleviate the stress of plating the Zn anode and hinder the extension of Zn dendrites, a zinc‐enriched liquid metal, ZnGaIn anode was coated on MXene layers using a blade coating approach (Figure 7f).^[^ ^72^ ^]^ This results demonstrates a significant reduction in the nucleation energy barrier and a decrease in the nucleation overpotential of zinc to 0 V versus Zn^2+^/Zn. As a result, the fabricated zinc‐based anode shows a long cyclic life up to 600 h and good high‐rate capabilities, with a current density of 8.0 mA cm^−2^ in symmetric cells.

## 4 Electrolyte Additive with MXene

**Figure 8:**
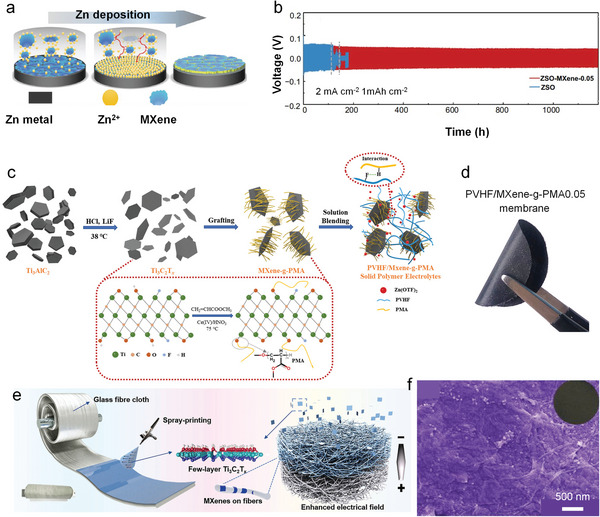
MXene as electrolyte additive and separator modifier. a) Schematic of improved Zn deposition induced by MXene additive, and b) the voltage profiles of Zn symmetrical cells with and without MXene additives. Reproduced with permission.^[^ ^77^ ^]^ Copyright 2021, Springer Nature. c) Schematic of the preparation procedure of solid polymer electrolyte, and d) photograph of the as‐prepared polymer electrolyte showing its mechanical flexibility. Reproduced with permission.^[^ ^79^ ^]^ Copyright 2021, Royal Society of Chemistry. e) Schematic of the preparation procedure and role of MXene‐GF separator. Reproduced with permission.^[^ ^80^ ^]^ Copyright 2022, Wiley‐VCH. f) SEM image of MXene@NiO modified separators. Reproduced with permission.^[^ ^81^ ^]^ Copyright 2022, American Chemical Society.

The use of electrolyte additives, such as dimethyl sulphoxide,^[^ ^75^ ^]^ methanol,^[^ ^56c^ ^]^ and saccharin^[^ ^76^ ^]^ is an effective way to suppress the dendrite formation. However, the electrolyte‐anode interface induced by electrolyte additives generally has a low ion conductivity, which potentially hinders the uniform nucleation of Zn ions. MXenes with high ion conductivity is applied as an electrolyte additive which is proven effective in improving the reversibility and kinetics of Zn plating/stripping. Through electrostatic interaction, Zn^2+^ and MXene additives may mix to form a conductive buffer layer on the surface of a Zn electrode (**Figure** **8**a).^[^ ^77^ ^]^ The MXene‐Zn^2+^ functional layer serves to homogenize the dispersion of Zn^2+^ on the surface, providing evenly distributed “seed spots” that promote uniform nucleation and ensure a homogeneous flow of ions during deposition process. Furthermore, the presence of MXene sheets in the electrolyte significantly reduces the diffusion pathway for Zn^2+^, enhancing their migration and improving the kinetics of the plating/stripping process. Therefore, MXene‐comprising electrolyte facilitates free‐dendrite Zn anode with 99.7% coulombic efficiency and enhanced reversibility (maintained up to 1180 cycles). (Figure 8b). The Zn‐V_2_O_5_ cell demonstrates enhanced cycling performance, improved cycling stability, and high coulombic efficiency when using an electrolyte containing MXene.

Additionally, TiO_2_ nanosheets derived from Ti_3_C_2_T*_x_* MXene were used as an additive in gel polymer electrolyte (GPE) for Zn‐ion batteries.^[^ ^78^ ^]^ The conversion of Ti_3_C_2_T*_x_* MXene into TiO_2_ nanosheets was achieved through a one‐step hydrothermal reaction, allowing for adjustment of the nanosheets structure and composition by controlling the heating conditions. The optimized TiO_2_‐containing GPE based on polyvinyl alcohol exhibits increased ionic conductivity and improved mechanical properties. This enables enduringly stable and reversible Zn plating/stripping for over 3000 h in PVA‐Zn (CF_3_SO_3_)_2_‐3% TiO_2_(PZ3T) GPE. Moreover, the full cell using the optimized GPE shows high capacity (216 mAh g^−1^ after 115 cycles) and good cycling stability. Furthermore, a solid polymer electrolyte (SPE) relying on poly(vinylidene fluoride‐co‐hexafluoropropylene) filled with poly(methyl acrylate) linked MXenes (PVHF/MXene‐g‐PMA) to address passivation and hydrogen evolution reaction side reactions (Figure 8c,d).^[^ ^79^ ^]^ The incorporation of grafted PMA into the PVHF matrix results in the uniform dispersion of MXenes, significantly enhancing the conductivity by three orders of magnitude compared to the PVHF matrix alone. And dendrite‐free Zn plating and stripping with high reversibility is accomplished, demonstrates by over 1000 h of cycling under ambient conditions and 200 h at elevated temperature. Furthermore, solid‐state full cell utilizing this SPE showcases outstanding cycling performance with 10 000 cycles at 2C under ambient conditions, and reliable operation within a wide temperature range (−35 to 100 °C). Notably, the all‐solid‐state Zn‐ion batteries demonstrate good shelf life, maintaining stability for over 90 days after storage at low and high temperatures.

## 5 Separator Modification by MXene

Separator is the key component of rechargeable batteries with the primary function of preventing an internal short circuit by separating the cathode and anode while permitting ion passage. It has been demonstrated that decorating the separator with components can homogenizes Zn^2+^ effectively and inhibits the production of Zn dendrites. For example, a Ti_3_C_2_T*_x_* MXene‐decorated Janus separator was developed via spray‐coating MXene nanosheets onto the surface of commercial glass fiber substrate. The MXene‐decorated separator shows enhanced surface polar groups, electrolyte wettability, and ionic conductivity, leading to improved local current distribution and Zn nucleation kinetics, as shown in Figure 8e.^[^ ^80^ ^]^ With MXene‐decorated separator, symmetric cell demonstrates dendrite‐free Zn anode and achieves stable cycling for nearly 1200 h at both 1 and 5 mA cm^−2^. Furthermore, an MXene/nanoporous NiO heterostructure‐engineered separator was fabricated by electrostatic attraction self‐assembly (Figure 8f).^[^ ^81^ ^]^ The MXene@NiO layer plays a crucial role in homogenizing the electric field distribution, reducing nucleation barrier for Zn deposition, and facilitating ion migration by minimizing the Zn^2+^ concentration variation. This results in uniform Zn plating, suppressing side reactions, and improving long‐term cyclability, and shows stable polarization for 2400 h at 2 mA cm^−2^.

## 6 Conclusion and Outlook

**Figure 9:**
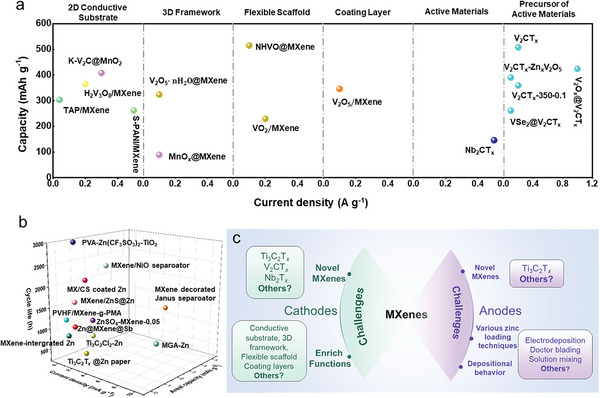
Summary of the electrochemical performance of functional MXene‐related studies in AZIBs. a) Cathodes and b) anodes, and c) outlook.

Recent research has demonstrated that MXenes offer a few advantages in AZIBs owing to their unique structural characteristics, such as high conductivity, structural tunability, mechanical stability, and various functional groups. The high conductivity of MXenes can ensure an efficient electron transfer to improve charge‐transfer kinetics and thus boost the rate capability and cyclability. The various surface functional groups on MXenes can serve as the active sites for constructing heterogeneous interface with the electrode material. According to prior studies, this heterogeneous interface may be inducive to improving electrochemical kinetics due to facilitated electron/mass transfer. For cathodes, MXenes and their derivatives can be used as a 2D conductive substrate, 3D framework, flexible scaffolds, coating layers, active components, and precursor of active materials, as exemplified by Ti_3_C_2_T*_x_*. The V_2_CT*_x_* oxidation derivatives and Ti_3_C_2_ MXene with surface functionalities like Cl, Br, and I have been used as active materials of AZIBs. The Nb_2_CT*_x_* MXene exhibits voltage plateau characteristics when tested in a high‐voltage region. In addition, MXene can operate as a host with a conductive skeleton or specific termination or mixed zincophilic seeds to increase the plating/stripping of zinc ions. In addition, MXene may be used as an additive of aqueous electrolytes, an inorganic filler of solid electrolytes, and a modifier of separators in solid zinc batteries. These all can effectively reduce corrosion and dendrite formation of zinc metal anodes. Despite the large number of reports, the application of MXenes in AZIBs is still in its early stage, and the development of high‐performance zinc ion energy storage devices encounters the challenge of scalability. In this paper, we have summarized and discussed recent studies on functional MXene‐related materials in both cathode and anode of AZIBs (**Figure** **9**a,b) to guide researchers for rational design of functional MXene‐based materials. The major challenges and possible development in the MXenes are summarized as follows (see also schematics in Figure 9c):

*Develop new MXenes with high activity and enriched functions in AZIBs cathode*: Currently, there are mainly three types of MXenes that are used as the cathode material for AZIB: Ti_3_C_2_T*_x_*, V_2_CT*_x_*, and Nb_2_CT*_x_*. Among them, Ti_3_C_2_ (‐O/‐OH/‐F) is a widely studied MXene member that provides conductive pathways in composite electrodes. However, during the synthesis stage, surface oxidation needs to be avoided to maintain high electronic conductivity of the composite material without sacrifice in its electrochemical performance. V_2_CT*_x_* and Nb_2_CT*_x_* exhibit some zinc storage activity, but their specific capacities are relatively low and need to be improved. Therefore, the exploration for MXenes with high zinc storage activity is crucial for developing high‐performance MXene‐based cathode materials for AZIBs. Possible routes and associated challenges are: i) Green and safe synthesis routes for large‐scale production of MXenes; ii) Exploring new MXene compositions in AZIBs, such as with transition metal carbides; In the cathode of AZIBs, the structural roles of MXenes in the composites are restricted to the 2D conductive substrate, 3D framework, flexible scaffold, and coating layers. Expanding the functional versatility requires deep‐dive research and understanding of the mechanism behind their multifunctional effects. iii) MXenes are derived from the etching of MAX phases. The recent success in utilizing the “chemical scissors” approach for structural engineering of MAX phases has brought new opportunity to develop functional MXene materials. The application of such functional MXenes in the AZIBs cathodes is yet reported but certainly worth investigating.

*New MXenes host for zinc loading and protection*: A high performance of zinc metal anode requires dendrite‐free zinc deposition, inhibition of side reactions and good reversibility, as well as high utilization rate of anode (DOD >40%). While MXenes have been proven useful in stabilizing Zn anode, it is still challenging to solve the multiple issues of zinc anode in one go. At present, only the Ti_3_C_2_T*_x_* MXene has been employed in AZIB anodes. Other types of MXenes, such as high‐entroy ones, with similar structure but different composition or surface chemistry should also be explored. On the other side, large‐scale monolayer MXene could be a favorable choice for surface protection due to stronger interface adhesion which offers a buffer for volume change. However, fabrication of large‐scale monolayer has been a challenge. The major approach of zinc loading on MXene is by electrochemical deposition. This method is difficult to control the zinc loading and uniformity. Dry methods could be more facile and environmentally friendly.

Better ionic conductivity can be achieved by constructing MXenes‐based composite anodes. This is achieved by incorporating MXenes into the zinc powder matrix or creating MXene‐based coatings on the surface of the zinc electrode. It has been widely accepted that the distinctive 2D layered structure and exceptional electrical conductivity of MXenes contribute to improved ion transport within the electrode, facilitating the transport of zinc ions during plating/stripping processes. The enhanced ionic conductivity of the zinc anode promotes more efficient electrochemical reaction kinetics which benefit to suppress the dendrite growth of Zn.

While the current focus of utilizing MXenes in anode protection is to tackle the problem of zinc dendrite formation, another important function that has been expected for MXene is to suppress the gas‐evolution side reaction. HER is a critical problem in AZIBs as the generation of hydrogen gas through water electrolysis can lead to reduced battery efficiency, compromised safety, and damage to the battery. However, the use of MXenes to mitigate the HER issue has not been scarcely reported and there are so far no mature solutions available. In our opinion, we can learn lessons from the widely reported inorganic or hydrogel protection layers. Specifically, surface chemical modification of MXenes could be considered to enhance its bonding ability with Zn surface and interaction with solvents. MXenes possess a rich and highly tunable surface chemistry, allowing for the introduction of functional groups to enhance the desolvation capability. Such modifications may benefit the interactions between MXene and solvents, thereby mitigating the HER and corrosion issue of the zinc anodes. Theoretical calculation or molecular dynamic modelling can help guide the design. In addition, synchrotron based high‐precision probe of surface chemistry or in situ spectroscopic observation of ions movement on the surface of MXene could be an option to understand the functional role of MXene materials in mitigating the HER issue.

In our discussion on zinc surface modification by MXene, the role of MXene is a surface protection layer. However, it is not fully certain whether zinc is deposited directly onto the surface of MXene (since it is highly conductive), within the interlayers (since most MXene used are in nanosheet structure of multiple layers), or beneath the MXene layer. For a true protection function, Zn is supposed to be deposited between the protective layer and underneath Zn. Hence, this particular aspect should be further deep articulated. For example, the elemental distribution of can be mapped by conducting high‐resolution EDS examination of the cross section after different plating stages. Additional, MXene derivatives due to Ti oxidation will reduce the conductivity and may change its role in Zn surface stabilization. Elucidation of this point will be of important for mechanism understanding and material design.

Looking into the future, researchers are suggested to make more use of the rich physical and chemical properties of MXenes and deep explore their versatility to further promote the development of high‐performance AZIBs.

## Conflict of Interest

The authors declare no conflict of interest.
